# Sentiment Analysis of Image with Text Caption using Deep Learning Techniques

**DOI:** 10.1155/2022/3612433

**Published:** 2022-06-25

**Authors:** Pavan Kumar Chaubey, Tarun Kumar Arora, K. Bhavana Raj, G. R. Asha, Geetishree Mishra, Suresh Chand Guptav, Majid Altuwairiqi, Musah Alhassan

**Affiliations:** ^1^Department of Applied Sciences Engineering, Tula's Institute, Dhoolkot, Dehradun, Uttarakhand, India; ^2^Department of Applied Sciences and Humanities, ABES Engineering College, Ghaziabad, Uttar Pradesh, India; ^3^Department of Management Studies, Institute of Public Enterprise, Hyderabad, Telangana, India; ^4^Department of Computer Science & Engineering, B.M.S. College of Engineering, Bangalore, Karnataka, India; ^5^Department of Electronics & Communication Engineering, B.M.S College of Engineering, Bangalore, Karnataka, India; ^6^Department of Computer Science and Engineering, Panipat Institute of Engineering and Technology, Samalkha, Panipat, Haryana, India; ^7^Department of Computer Science, College of Computers and Information Technology, Taif University, Taif, Saudi Arabia; ^8^University of Development Studies, Electrical Engineering Department, School of Engineering, Nyankpala Campus, Nyankpala, Ghana

## Abstract

People are actively expressing their views and opinions via the use of visual pictures and text captions on social media platforms, rather than just publishing them in plain text as a consequence of technical improvements in this field. With the advent of visual media such as images, videos, and GIFs, research on the subject of sentiment analysis has expanded to encompass the study of social interaction and opinion prediction via the use of visuals. Researchers have focused their efforts on understanding social interaction and opinion prediction via the use of images, such as photographs, films, and animated GIFs (graphics interchange formats). The results of various individual studies have resulted in important advancements being achieved in the disciplines of text sentiment analysis and image sentiment analysis. It is recommended that future studies investigate the combination of picture sentiment analysis and text captions in more depth, and further research is necessary for this field. An intermodal analysis technique known as deep learning-based intermodal (DLBI) analysis is discussed in this suggested study, which may be used to show the link between words and pictures in a variety of scenarios. It is feasible to gather opinion information in numerical vector form by using the VGG network. Afterward, the information is transformed into a mapping procedure. It is necessary to predict future views based on the information vectors that have been obtained thus far, and this is accomplished through the use of active deep learning. A series of simulation tests are being conducted to put the proposed mode of operation to the test. When we look at the findings of this research, it is possible to infer that the model outperforms and delivers a better solution with more accuracy and precision, as well as reduced latency and an error rate, when compared to the alternative model (the choice).

## 1. Introduction

Sequentially using picture sentiment analysis in conjunction with other methods, the system may be able to evaluate photos and extract inner sentiments [1, 2] from them (when used in conjunction with other approaches). Due to the increasing number of people who express their views and feelings on the Internet [2], the automated evaluation of such emotions and sentiments is becoming more popular in the field of opinion analysis. This tendency has already been successfully implemented in several industries, including advertising, entertainment, and educational settings [3]. To study the views of the general population, previous methodologies emphasized level-1 aspects such as pattern, color, texture, size, and form [4] to assess their perceptions. In recent years, deep analysis of in-depth characteristics has become increasingly automated, thanks to the emergence of machine learning embedding approaches [5]. These procedures have been evaluated on large-scale training sets and be successful in most cases [6]. Many researchers have undertaken opinion analysis using deep learning algorithms in recent years [7, 8], and the findings of their work have been published in peer-reviewed publications as a consequence of their efforts.

The use of deep learning with CNN has shown its effectiveness in the challenge of image-based opinion categorization by proving the benefit of learning in-depth characteristics and executing the learning process in a real-world context. When dealing with large datasets [8, 9], the classification accuracy improves as a consequence of the increased processing power made accessible to the algorithm by the increased processing capacity [9]. Because it requires a significant commitment of both time and money, opinion analysis is usually seen as a difficult undertaking [10]. Also noteworthy is the fact that emotional objects are not self-sustaining and, as a result, are not easily accessible as a study resource [11].

Deep learning methods, which leverage a restricted set of independent sample attributes for training and testing, have been developed to overcome these concerns. Several studies [12] have shown that these techniques are quite accurate. [13] During the assessment phase, the ADL may analyze every occurrence of the features from the labeled sample data [14] by referring to the labels from the labeled sample data that were used to label the features in the sample data during the preceding assessment phase of the evaluation [15]. A deep learning-based model for effectively collecting sentiment information is presented in this article, which analyzes the attributes of an opinion before merging the information with other sources. The model is based on the active deep learning classifier and the map of the text content of the review with the image properties, and it is implemented in Python [16]. In addition to sending information about picture material and text content to a target audience, it is feasible to communicate information about both image material and text content using both DLBI and ADL data, which is an important advantage [17]. With the help of the DLBI, it is possible to map several convolution layers made of picture data and text captions to get precise information about the perspectives expressed on various social media platforms [18].

Sentiment analysis is used to identify individual actions, views, tests, and feelings regarding a person or group of persons, problems, behaviours, or subjects, as well as the features of such activities, views, tests, and emotions In our activity, opinions are important since they have the most influence on the results [19]. Typically, sentiment analysis is used to aid in the categorization of data by taking into consideration both the positive and negative aspects of a certain topic matter. As a result, it is possible to obtain both positive and worthy views about a certain product as a result of this, and people are appreciative of this [20]. Opinion mining is best suited for businesses and large organizations as a marketing tool.

Using SA (sentiment analysis), organizations may learn more about their customers' attitudes toward their products and services, and how they can improve their operations. As the characteristics of the items are identified, as is the identification of the primary source of less income, it becomes necessary to pay more attention to the diverse opinions of customers on those characteristics [21]. Opinion mining is one of the most useful tools available in the worlds of business, public relations, and reputation management, and it may be utilized to great effect in these fields. Keeping track of user activities allows the organization to keep sequence predictions working properly and efficiently.

Within this chapter, the process for efficient sentiment analysis is discussed, which is used to extract and categorize the views conveyed in text messages. This research endeavor is comprised of four major phases that are interconnected [22]. This step-by-step procedure includes data preprocessing, prospective feature extraction, producing opinions, and extracting and classifying opinions, among other things. To produce Part-of-Speech (POS) tagged text from datasets obtained from similar online publications [23], it is required to preprocess the datasets derived from comparable online publications. To get the potential features, an algorithm specifically created for this purpose (DLBI) enhanced high adjective count methods on the Part-of-Speech tagged text and applied them to the Part-of-Speech tagged text. DLBI efficiently advances the scores of words for them to acquire the potential qualities [24].

The DLBI method is supplemented with an AIBC (artificial bee colony) algorithm, which operates underneath the DLBI algorithm and provides rankings on opinions as well as ratings for each term. The opinion words are obtained via the use of a technology known as the Max Opinion Score [25]. A classification stage follows, in which the ID3 (Interactive Dichotomizer version 3) algorithm is used to divide the opinions into three categories: positive, negative, and neutral. The next stage is to analyze the data gathered throughout the research process. Experiment outcomes are compared to each other based on the data from the customer review dataset and other review datasets [25].

The remainder of the proposed work will be structured in the following way, by the pattern stated in the next paragraph: The mechanics of the different sentiment analysis models are discussed in detail in Section 2, while the findings of the relevant performance assessment are discussed in Section 3 and supplied in Section 4. Detailed results from the relevant performance evaluation are included in Section 3 of this report. Employing the VGG network-based intermodal sentiment analysis model, which is discussed in greater depth in Section 4, Section 3 delves into the difficulties of transferring the connection between words and pictures, which is discussed in greater depth in Section 4. Section 3 also delves into the difficulties of transferring the connection between words and pictures, which is discussed in greater depth in Section 4. Section 3 finishes with a discussion of potential study paths shortly (DLBI). An in-depth evaluation of the experimental data, as well as a discussion of the findings made by the researchers, may be found in this section of the article. It is possible to find the conclusion of this text in Section 5 of this document, which is placed after the publication.

## 2. Related Works

They conducted a study in which they evaluated three different Twitter sentiment analysis algorithms using three distinct metrics: pixels, stream, and themes (PST), according to the authors of [26]. To extract, map, and analyze user comments on an online product, the information acquired was put to use, and the findings were made available to the general public. A greater amount of attention was paid to negative factors as well as influencing variables than had been paid to them before [27]. When it comes to the analysis of the essential characteristics, there are no details, which represents a huge gap in our inquiry [28].

A weakly-supervised coupled convolutional network (WSCCN) was created to extract localized information from the collected opinion features, which was later published in their publication [17]. To get a deeper knowledge of human emotions, it is critical to use feature mapping techniques. There were certain limitations in that research, including the inability to do a correlation analysis across both text and images, which was also a limitation of our work [29].

Using a transfer learning technique, the authors of [18] constructed a visual sentiment analysis framework (VSAF) to predict customers' feelings, which they then put into practice. The system was given the moniker “Visual Sentiment Analysis Framework” by the researchers [30]. The use of this strategy has shown to be beneficial in the event of overfitting in the past. Despite extensive examination, the system is unable to finish the feature collection appropriately [28].

In this scenario, it has been proposed that the use of a deep coupled adjective-noun neural network might be an appropriate approach (DCANN). Specifically, my study [31] investigates the inner-class variance and the middle-level variance analysis in more depth within the framework of the opinion mining concept. It is possible to improve the performance of a prediction network by altering the parameters of the network [32]. Corrections can be applied to improve the prediction network's performance. Regarding sentiment prediction, the lack of independent middle-level analysis is to blame for the approach's lack of performance.

In Ref. [20], the authors discuss their research on machine learning algorithms for creating Emotional Arcs (ML-EA), to enhance the ability to predict audience engagement during presentations. Even though the outcomes of the tests are analyzed, the real-time application of the suggested approach is not considered [33].

According to the study's findings, there are severe faults in picture sentiment analysis and classification procedures, as shown by the survey's results, which support these conclusions. As part of this inquiry, DLBI is being utilized to solve challenges that have not previously been addressed, and it is now in the process of being deployed. Using DLBI, it is possible to transmit the relationship between the words and the visuals directly from the words to the images [34]. Using the VGG network software, opinions can be expressed numerically as vectors and then converted into a mapping process. Active deep learning (ADL) classifier must be utilized in order to accurately forecast views based on the information vectors acquired [35].

The approach that will be employed in the proposed research, if one is to be successful.

By mixing natural language processing (NLP) and artificial intelligence (AI) models, the basic goal of this research is to identify fake news in Twitter data. This will be done by combining NLP and AI models [36]. Since the advent of contemporary technology, which enables anybody to readily write news and spread it extensively via social media platforms, fake news has grown in importance. To achieve this aim, the proposed system employs a combination of natural language processing (NLP) and artificial intelligence (AI) approaches to recognize manufactured news items in the context of tweets from Twitter. Researchers believe that the collection included 3000 tweets based on Donald Trump's proposed works, which they used to create their findings [37]. An original Python script created expressly for this purpose was used to gather and analyze tweets for the project. The dataset includes tweets that were originally posted and were later determined to be either fraudulent or legitimate by a third party [38].

It is possible to extract perceptions from the WWW (World Wide Web) with the use of traditional data mining methods and transfer them as characteristics of the website via a process known as web mining [39]. The Internet might be regarded as the most essential channel for distributing and promoting information in the modern world. E-commerce websites, on the other hand, are intended to serve as vital marketing instruments for the business's activities. The Internet might be regarded as the most essential channel for distributing and promoting information in the modern world. E-commerce websites, on the other hand, are intended to serve as vital marketing instruments for the business's activities. When analyzing people's behaviour on a website, it is necessary to conduct data mining methodologies to understand their behaviour [40] better.

However, because of the diversity of ideas and the differences in their contents, the effective recovery of views continues to pose difficulties for both individuals and organizations. As a result, computer-assisted extraction and summary of opinions become more important [41]. As a result, when it comes to mining characteristics, opinion mining is shown to be the most powerful technique for mining the perspectives of goods from various websites. Furthermore, the rating based on customer opinions not only helps the client in understanding the goods, but also aids in the differentiation of the products into several various categories [42].

When developing the features for this study effort, opinion mining is used to gather information from different consumers about the items that are accessible on various websites, which is then used to develop the features [43]. The opinions expressed by users and their ratings for the product not only help consumers in gaining a better understanding of the item's quality but also aid in the differentiation of various things. This satisfies the criteria by completing two significant tasks [44]. Feature identification, on the other hand, is the work of deriving and locating characteristics from the views of the user, and it is the first step [45]. A further prediction is *L* (rating prediction), which examines the arithmetic ranking of the product characteristics. The ultimate goal of this project is to create a system for generating perspectives from the viewpoints stated by users in online forums and discussion boards.

## 3. Proposed Methodology

It is intended that the design of this investigation be divided into four major stages, which are as follows:

- Data preprocessing and preparation
- Possibility of feature extraction
- Developing and extracting views and information
- Classification of points of view

The data that are obtained from the web content are not appropriately formatted at the time of extraction. The first step prepares the data in preparation for the sentiment analysis and extraction to take place later on [44, 45]. Users' opinions in product evaluations are mined for product attributes using opinion mining as one possible method of discovering and matching up to the power and scarcity of things. An important step in the process of opinion mining is the extraction of features from the opinions of the participants [42, 43]. The system takes these characteristics as input, assigns them rankings, and then assesses whether the feature is good, negative, or neutral at the end of the process.

The DLBI characteristics are determined by using the method that has been provided. The ABC optimization method is responsible for this. This program generates a ranking for all nouns by evaluating every review [40, 41]. When determining whether scales are higher or lower than the threshold, the ranking concept is used.

A total of three primary inputs are required by the algorithm. The following are examples of what I mean:

1. A collection of adjectives that are used to express one's point of view
2. A numerical score indicating the degree to which an opinion is positive or negative
3. The collection of possible characteristics

These may be detected by the use of an algorithm such as DLBI. Finally, the estimate may be employed for this suggested algorithm's feature extraction from the many review sites, which will allow it to be more accurate. The newly presented framework divides the world into four categories [46, 47].

In addition to providing a good and negative evaluation, it also extracts substantially typical qualities for each evaluated item and assigns opinion rankings to each of these attributes, among other things. The last step will be completed with the assistance of a decision tree classifier that will make use of derived characteristics [2, 48].

### 3.1. Data Preprocessing

The initial phases of the project include gathering data from many online publications that will be used as inputs for the proposed technique. The inadequately structured or not structured data sources are collected, and they may be used for data processing and data storage in addition to being used for data collection [39, 48].

Raw input that is not adequately organized or inappropriate for further processing is treated with the appropriate technique of preprocessing, and the result is used for further processing [37, 38]. It is customary to undertake preprocessing in three major processes, which are as follows:

- Elimination of stop words
- Stemming process
- Representation of point of sale

After all of these steps are completed, a list of acceptable Part-of-Speech terms is generated from all documents. This is accomplished by deleting stop words and stemming the words in each document [35, 36].

### 3.2. Removing Stop Words

In the English language, there are around 400 to 500 stop words per thousand words. The use of stop words, such as a, an, the, is, was, and too, among others, is prohibited. The inadequate words are deleted as a result of reducing human flaws.

It is the way of obtaining root words from the consequent words that are acquired as a consequence of the process of deleting stop words that are referred to as “stemming.” Stemming may also be defined as the process of removing morphological and flexional ending words from a sentence or phrase [33, 34]. The process of stemming transforms the words into their corresponding stems.

According to this theory, words with the same word roots (or “stems”) convey concepts that are the same or extremely similar across texts, and hence, stems can be used to represent concepts in texts [32]. In this situation, nouns such as using, used, user, and users are all derived from the word “use.” In this scenario, nouns such as using, used, and users are all derived from the word “use.”

In line with the subject matter in which they appear, POS (Parts-of-Speech) tags are allocated to the words in which they appear. It is necessary to utilize WordNet to represent all of the sentences that are different from each other [28, 30].

One of the English lexical databases, WordNet was built at the University of Princeton under the direction of Ref. [31] and made accessible on the Internet. Synsets such as verbs, adverbs, nouns, and adjectives are arranged cooperatively in the database to organize the category of cognitive synonyms, in which each synset provides a separate thought, and in which each synset presents a distinct notion, in which each synset provides a separate thought, in which each synset presents a distinct notion. The synsets are a collection of related terms [28, 29].

Depending on the links between lexical, conceptual, and semantic words, this may be accomplished. When it comes to dealing with the words of natural language, WordNet may be considered both an ontology and a knowledge base. This document has more than 100,000 words, which are organized in a taxonomic manner [26, 27].

As the name implies, synsets (also known as synonym sets) are collections of verbs, adverbs, nouns, and adjectives that have semantically related meanings [24, 25]. The form of senses is made up of a series of similar phrases or concepts that are distinguished by the use of various synonyms. Certain synsets have a stronger or weaker reciprocal interaction depending on the degree of connectedness between them.

When two distinct approaches to representing a connection are used, the results are usually more accurate [22, 23]. There are two types of synonyms: hyponyms and hypernyms, which indicate they are components of a relationship, and mers and homonyms, which mean they are components of a link.

It is discovered that the hyponym/hypernym is the secondary organizing force in the system. An example, when one phrase is referred to as the hyponym of another term, the preliminary word has a more different meaning when compared to the final term, in which case the final term is referred to as the hypernym of the previous one [20, 21]. There are transitive and contradictory relationships between two or more words, which are shown in this sentence.

Except for adverbs and adjectives, the hyponym/hypernym has a larger number of verbs and nine nouns than the average word. In the case of ontologies such as Open Directory, their synset recognition may be altered if they are provided in a newer version than the one that was previously released [18, 19]. Although the backward compatibility utility program may be used to show synsets across multiple versions of the software, it is not necessary for this instance. The document containing product reviews is also separated into several text files, each of which includes a single review for a different product. To make the content more manageable, this is done. According to input data provided throughout the processing procedure, the preprocessing technique generates a textual output that is marked for each portion of speech [16, 17].

A DLBI algorithm is then used to the tagged text of Part-of-Speech to obtain the prospective characteristics for which a DLBI method is used [14, 15]. Among the algorithms in the DLBI were two of the most important: They are as follows:

- Algorithm with a High Adjective Count (HAC)
- The artificial bee colony (ABC) algorithm is a kind of algorithm.

HAC is the name given to the algorithm that is used to find the most likely characteristics. This HAC extracts the primary fundamental qualities that allow the opinions of the users to be presented clearly and concisely [12, 13]. Nouns are considered to be the most important Part-of-Speech tag since they may convey the characteristics of a certain product. Typically, the vast majority of studies adopt phrase frequency as a method of keyword selection [10, 11]. This intended strategy, which is an alternative to the term frequency approach, makes use of nouns and adjectives in conjunction with the POS tag of a review document.

When it comes to this HAC method, adjectives are considered to be the most essential factor, and the sum of all adjectives makes it easier to discover the perspective characteristics [8, 9]. All of the reviews are dealt with one by one to determine the relative importance of the various points of view. From every point of view, the procedure is carried out sequentially. The nouns and adjectives are found at the moment of the process's inception, as are the ranks of each noun, and the sum of each adjective is set to zero at the start of the process [6, 7]. In the case of an adjective that is extremely close to the particular noun, the rank of the noun is increased by one. As a result, for each noun in each review of the collected input database, the scores that are referred to as the opinion scores are assigned to it [4, 5].

The higher-ranking nouns are those that have a greater number of adjectives attached to them than the lower-ranked nouns. The duty of a ranking noun is completed by the opinion ratings assigned to each noun in the ranking. Nouns are separated from one another in this activity of ranking, which is followed by the selection of possible qualities from the class of nouns [2, 3]. This method has a threshold that must be met for the probable characteristics to be successfully deduced.

Utilizing improved HAC, the noun scores may be efficiently maximized to acquire the feasible features, which are then assessed. It is vital to optimize the hierarchy after establishing the order of the points of view, and all nouns should be rated using the ABC approach to do this.

**Figure 1:**
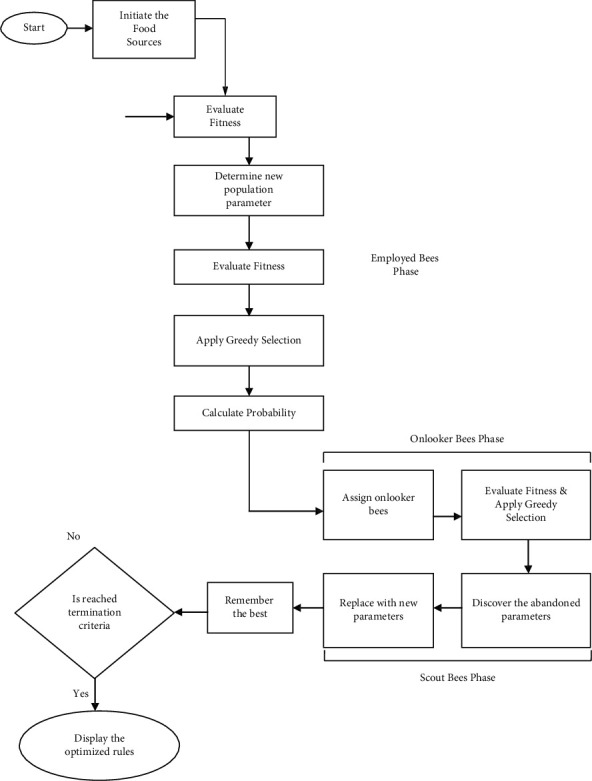
Flowchart for the ABC algorithm.

It includes the three key elements, which are specially employed bees, observer bees, and scout bees, as well as other bees. They are associated with food sources within a short distance of their hive, and they provide information to observer honeybees about the nectar content of food sources they have used in the recent past. Onlooker bees are watching the employed bees dance within the hive to choose which food source to utilize based on the information provided by the employed bees and the data provided by the employed bees. If the food sources of the employed bees are no longer being utilized, the bees transform into scout bees, who look for new food sources at random. Food sources show the location of alternative solutions to optimization issues in terms of their quantity, and the total amount of food sources reflects the aspect of the solution in terms of its aspect. The ABC algorithm's working method is shown in Figure 1.

#### 3.2.1. Preliminary Phase

Primarily, a population of the food origin *x*_*i*_, (*i* = 1, 2,…, *R*) is created at random, in which *R* indicates the population size. This food origin comprises the value of the index and its relative values of rank (*R*_*i*_), which are produced for all nouns. This task of generation is referred to as the process of initialization. To compute the finest food source, the fitness value of the created food origin is evaluated using the following equation:

(1) $$\begin{array}{c} \text{Fitness Function},\left(i\right)=\text{ma}\left(\text{Rank}\right). \end{array}$$

After evaluating the fitness value, the iteration is fixed to one.

Afterward, the stage of the employed bee is achieved.

(2) $$\begin{array}{c} V_{i,j}=x_{i,j}+\phi_{ij}\left(x_{i,j}-x_{k,j}\right), \end{array}$$

where *k* and *j* are indices that are chosen arbitrarily, *φ* denotes the number generated at random in the range [−1, 1], and *V*_*i*,*j*_ is the *j*^th^ position new value and then evaluated for all newly created population parameters of food origin. From the evaluated value of fitness of the population, the finest parameter of the population is chosen, that is, the population parameter that possesses the best value of fitness by implementing the process of greedy.

When determining the number of observer bees, it is necessary to determine the probability of the given parameter being fulfilled. It is defined by the probability value *P*_*j*_ how quickly new solutions *V*_*i*,*j*_ for observer bees are made from the solutions *x*_*i*,*j*_ when new solutions *V*_*i*,*j*_ for observer bees are formed from the solutions *x*_*i*,*j*_. The fitness of the function concerning the new solution is checked in the next phase, and the technique of greedy selection is carried out later on to choose the optimal parameter.

At this stage of the procedure, certain elements that were not taken into consideration by the scout bees are identified. It is assumed that any unutilized parameter values exist, and if so, they are changed by new parameters chosen by scouts using equation (3.3), and the value of fitness is calculated using the newly picked parameters. The perfect configurations have been preserved in the system for the time being. As the number of iterations rises, the technique is repeated until the ending condition is reached. After the method, the best rankings and their relative indices are established.

As a result of the ABC approach, the optimum scores are created, which are then utilized to derive the prospective features by comparing the score values with a predetermined cutoff. To detect any probable qualities of the data, the algorithm ABC is used once this step is completed. Therefore, the DLBI approach is used to gather the potential attributes that are necessary.

The opinion words are generated by detecting the potential attributes, and the mining process is carried out with the help of the Max Opinion Score Method, which is a machine learning algorithm that is used to generate the opinion words.

Those adjectives that are near to the noun, as well as any derived prospective attributes, are provided as inputs to this technique. They are referred to as opinion words since they are made up of a collection of adjectives that are used to indicate different points of view. Following that, the ranks are awarded to each adjective in this collection that has characteristics that may be classed as either good or negative, as shown by the rankings table. The rankings are awarded based on the terms that are used to represent an individual's point of view. It is clear from the negative rankings that the perspective is represented via the use of negative opinion phrases and vice versa. Positive opinion words have higher values than negative opinion words, while negative opinion words have lower values than positive opinion words. Positive opinion words have higher values than negative opinion words.

Inversion words may change the meaning of a word's expression by modifying the sensitivity of an opinion word when compared to the sensitivity of other opinion terms in the same group. There are a few examples of inversion word use, such as the terms “un,” “not,” and “non.” For example, the word “great” is a positive term that shows that the speaker has a good attitude about something. In this case, the phrase “not good” occurs before the positive opinion word and is followed by the inversion word “not,” which indicates that it is a negative opinion. This is why the point system is situated on the left-hand side of the page when it comes to giving points for words expressing one's viewpoint. Whenever any inversion words are identified as opinion terms, the score of any inversion words that have been categorized as opinion terms is deducted by a factor of one. As a result, points are awarded to words that indicate a change in point of view.

The inputs for the matching process are considered to be the opinion words that are located near the derived potential attributes, and the derived potential attributes are considered to be the outputs of the matching process. To discover the potential qualities, it is important to use the DLBI methodology. Almost every one of the evaluations is based on how well the sentences are constructed. For each phrase, the opinion words and prospective characteristics that are the most similar to all of the opinion words are identified and determined, as is the relationship between the opinion words and prospective qualities, as well as the relationship between the opinion words and prospective qualities.

For every conceivable detail under investigation, the scores for all possible qualities are calculated by taking into account each word's opinion score and summing it up. As a consequence, the review rating is calculated by adding up all of the ratings that have been given for the whole range of available characteristics. Final calculations are made to determine the average rank for each opinion word for each possible attribute, and the findings are presented in a tabular format. Here is how to generate pseudocode for the whole process of opinion generation and mining, which includes the Max Score technique (Algorithm 1):

### 3.3. Classification of Opinion

**Figure 2:**
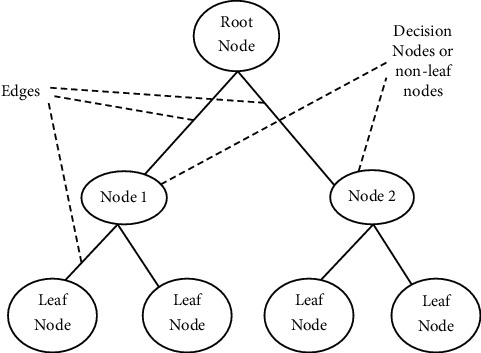
Structure of decision tree.

This phase is required for the organization of reviews that include three different sorts of product opinions. A method of classification known as the ID3 algorithm may do this. It is one of many classification algorithms available. It is possible to categorize reviews into three categories: good, negative, and neutral, depending on the organization that organizes the opinions. These three categories are related to different points of view, Figure 2.

According to this research, the classification's result may be any one of the following three outcomes:

The following is the intermediate information that is required to create the decision tree as an origin of result PR, NR, or NTR:

According to equations (3)–(5), the weight for the *i*th branches is equal to the amount of the classes *T*  that integrate to the value of *T*_*i*_. The value of information gain IG may then be determined by branching on *X*_*i*_, as demonstrated in the example below:

(3) $$\begin{array}{c} \text{IG}\left(X_{1}\right)=I\left(a,b\right)-\text{EI}\left(X_{1}\right), \end{array}$$

(4) $$\begin{array}{c} \text{IG}\left(X_{2}\right)=I\left(b,c\right)-\text{EI}\left(X_{2}\right), \end{array}$$

(5) $$\begin{array}{c} \text{IG}\left(X_{3}\right)=I\left(a,c\right)-\text{EI}\left(X_{3}\right). \end{array}$$

The ID3 method examines every feature *X*_*i*_, and the *X*_*i*_ that provides the greatest amount of IG (*X*_*i*_) value is selected for use in the construction of the decision tree.

After that, the same procedure is repeated over and over again for each of the remaining subsets *T*_1_, *T*_2_,…, *T*_*N*_ to construct the tree. If every class is positive for all *T*_*i*_ (*i* = 1, 2,…, *N*), then it chooses with the “YES” node and finishes the process; if every class is negative, then it decides with the “NO” node, and the process is terminated. Otherwise, ID3 picks one additional characteristic similar to that which was supplied before, resulting in the neutral class being created with a “neutral” node. Customers' opinions are used to categorize the reviews, which are then divided into three categories: good, negative, and neutral.

## 4. Results and Discussion

The outcome of the suggested implementation is shown and discussed in depth in this section, which includes the findings of experimentation.

To assess the efficacy of opinion mining systems and to explain both the hypothetical and real evolution of these systems, an evaluation metric is developed and used. It consists of a set of measurements that are trailing behind the overall assessment approach that has not been released. Recall, precision, and the F-measure are just a few of the metrics that have been used for assessment and testing. It is necessary to compute the values of these measures to make better use of this suggested approach for the efficient categorization of reviews using mining to make better use of it.

**Figure 3:**
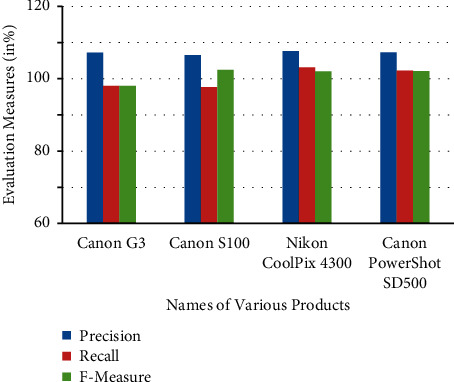
Evaluation measures of precision, recall, and F-measure for various products of proposed work.

According to the review classification chosen, the values of measures are calculated in Figure 3.

**Figure 4:**
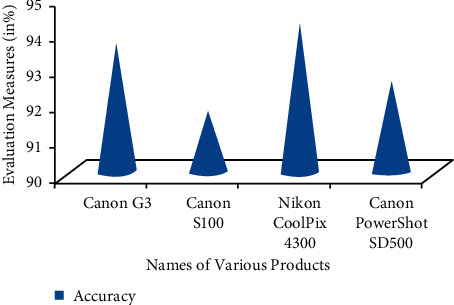
Performance metrics.

The product evaluations of numerous firms are obtained in this study utilizing the DLBI approach, which is described in detail below. The product is being evaluated in conjunction with various types of particular things at this moment. The Canon S100, Canon PowerShot SD500, Canon G3, and Nikon Coolpix 4300 are among the goods that have been reviewed. Various sorts of camera devices may be used to evaluate the review performance of suggested work, and they are listed below (Figure 4).

To facilitate comparisons across estimates for the same product from several different businesses, we recommend that you use the DLBI technique described above.

The values of TP, FP, TN, and FN values may also be described to get the accuracy measure values. If you look at these different sorts of camera goods side by side, the Nikon Coolpix 4300 model reaches the maximum degree of precision (94.24 percent). 93.05 percent is the average grade achieved by the planned task, according to the grading scheme. When machine learning methods are used, this results in very accurate opinion mining and review classification when the data are analyzed. Therefore, the DLBI algorithm presented here produces opinions from Internet users in the right manner and categorizes their reviews according to their categories.

## 5. Conclusion

In this research endeavor, the DLBI technique for opinion mining is described in great depth in detail. The overall performance of DLBI was evaluated based on the product reviews collected. Precision, recall, accuracy, and the F-Measure were determined for each of these assessments and then compared to one another to see which was the most accurate. Additionally, the findings of the comparison of existing procedures with DLBI, as well as the accuracy values connected with each method, are presented in this proposed work.

DLBI-based opinion mining and classification methodology are compared to prior methodologies for opinion mining and classification offered in the literature review, as well as to the suggested DLBI-based opinion mining and classification methodology.

DLBI and ID3 mining and classification procedures are recommended, and it achieves around 94.45 percent precision for these techniques, which is a high degree of accuracy for these approaches.

The average utilization of both DLBI and ID3 is shown, indicating the efficacy of both techniques. The work outlined in this proposal contributes to the improvement of the process of opinion mining and classification. Two additional contemporary algorithms, J48 and Bagging of state-of-the-art works, reach accuracy values of just 88.51 percent and 87.5 percent, respectively, when compared to the accuracy value.

## Figures and Tables

**Algorithm 1:**
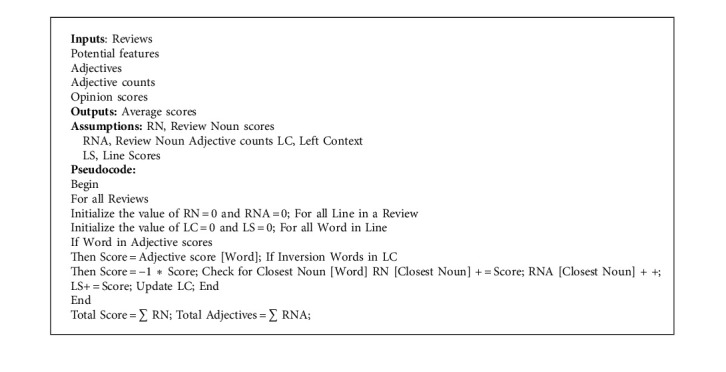
Pseudocode for max opinion algorithm.

## Data Availability

The data that support the findings of this study are available from the corresponding author upon request.
